# Temporal evolution of the Mediterranean fin whale song

**DOI:** 10.1038/s41598-022-15379-0

**Published:** 2022-08-09

**Authors:** Paul Best, Ricard Marxer, Sébastien Paris, Hervé Glotin

**Affiliations:** 1 Université de Toulon, Aix Marseille Univ, CNRS, LIS, DYNI, Marseille, France; 2 Pôle INPS, Marseille , France

**Keywords:** Marine biology, Acoustics, Computer science

## Abstract

We present an analysis of fin whale (*Balaenoptera physalus*) songs on passive acoustic recordings from the Pelagos Sanctuary (Western Mediterranean Basin). The recordings were gathered between 2008 and 2018 using 2 different hydrophone stations. We show how 20 Hz fin whale pulses can be automatically detected using a low complexity convolutional neural network (CNN) despite data variability (different recording devices exposed to diverse noises). The pulses were further classified into the two categories described in past studies and inter pulse intervals (IPI) were measured. The results confirm previous observations on the local relationship between pulse type and IPI with substantially more data. Furthermore we show inter-annual shifts in IPI and an intra-annual trend in pulse center frequency. This study provides new elements of comparison for the understanding of long term fin whale song trends worldwide.

## Introduction

The fin whale (*Balaenoptera physalus*) is commonly found in the Mediterranean Sea, with an estimated population of approximately 3500 individuals in the Western basin^1^. As cetaceans, they are highly vocal animals, making the most out of the favorable underwater sound propagation (especially compared to light propagation). Their vocalizations, are low frequency sounds, such as 40 Hz down-sweeps, 30 Hz rumbles, and 20 Hz pulses^2^. The latter, sometimes also referred to as call or note, is termed pulse for its relatively short and transitory nature. In general, fin whale vocalize supposedly for group cohesion^2,3^, food signaling^4^, and mate attraction^5,6^.

This study focuses solely on the sequenced 20 Hz pulses of the fin whales. The stereotyped patterns in these sequences, as well as their potential reproductive function have motivated terming them as ‘songs’^5^. The function(s) of songs in animal communication systems are commonly described as a means of territorial defence, mate attraction and/or mate selection^7^. Songs may convey information about individual’s physical and cognitive fitness^8^ (via the pitch or the ability to learn and reproduce sequences). While the function(s) of the Mediterranean fin whale songs may be the same as those in other oceans, their structures vary.

Indeed, as in other cetacean species, fin whales show geographical acoustic differentiation in their songs^9–12^, hypothesised to be cultural in some cases^9,10^. The Mediterranean population, shown to be resident and genetically dissociated from the North Atlantic population^13^, has a specific song structure that enables its acoustic identification^12,14^. Additionally, it is worth noting that Mediterranean fin whales do not follow strict migration patterns or reproduction periods unlike their oceanic conspecifics^1^, suggesting that their song could be heard all year round (for other populations, songs are heard only during the reproductive season^2^).

**Figure 1:**
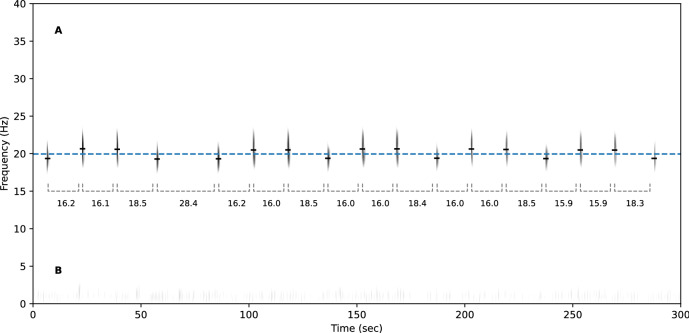
Spectrogram of a fin whale pulse sequence recorded by the Bombyx buoy in October 2018. Spectrogram parameters are described in section "Spectro-temporal pulse analysis". Dashes show the center frequencies of the detected pulses, with grey dashed lines showing the IPIs. The blue dashed line denotes the discrimination threshold between A and B pulse types, at 20 Hz.

The base unit of the songs, the 20 Hz pulse, is shared by all fin whales. These pulses occur in sequences that typically last several hours, with highly regular pulse intervals between 10 and 40 s^5^. The main differentiation of songs across populations lies in the IPI (sometimes called INI for Inter Note Interval) and pulse spectra^15,16^. Alike fin whales of the Pacific^9,10,17^, Mediterranean 20 Hz pulses fall into 2 distinct types, one with a slightly higher frequency content than the other^18,19^ (see Figs. 1, 5). These two categories are sometimes labelled classic 20 Hz pulse and back-beat, we will refer to them as type A and B for short (A being the higher pitched pulse). Fin whales of the Pacific and Atlantic often exhibit sequences that alternate between two stereotypical IPIs^9,10,20^. These are called doublet patterns, as opposed to singlets where only one IPI occurs. In doublets there is sometimes a strong relationship between IPI and pulse type: there is one IPI from A to B, and another one from B to A^10,11,21–23^. On the other hand, singlets also follow their own stereotypical IPI. Mediterranean fin whale songs show more diversity in the sequencing of pulse types than simple singlets or doublets (Fig. 1). Two studies present local stereotypical IPIs. Based on recordings from 1999, Clark et al.^18^ revealed a link between pulse type and IPI from two pulse sequences (about 100 pulses). About ten years later, Castellote et al.^12^ observed a common IPI around 14.9 s for that same population, but do not mention its relationship with pulse types.

Besides geographical variations, fin whale song structures also exhibit temporal variations, such as seasonal IPI increases^5,11,21^, and inter-annual variations of IPI and peak frequency^9,10,20,24^. Seasonal IPI increases appear to be synchronised with mating cycles, suggesting a link between the two (increasing testes activity or decreasing competition^21^). This highlights the importance of considering the song’s function in the interpretation of temporal patterns^25^. On the other hand, the drives for inter-annual trends remain unclear, with some emerging hypothesis for a cultural phenomenon^9,10,20^. Besides, with the observation of synchronous inter-annual shifts of both IPI and center frequencies in Pacific fin whale songs, the hypothesis of a link between the two arises. Weirathmueller et al.^9^ state that the augmentation of the IPI through the years could be explained by the simultaneous decrease in pulse centroid frequencies (lower frequency pulses presumably requiring a bigger effort to produce, a bigger gap between them could be needed). Inter-annual trends are also found in blue whales (not in call rate but rather in call frequency) but also lack an agreement among numerous hypotheses for their cause^25^ (e.g. cessation of commercial whaling^26^, increase in calling depth^27^, augmentation of noise from melting icebergs^28^ or acidification of the oceans^29^).

Passive acoustic monitoring (PAM) stations combined with automated analysis play a key role in revealing these long-term trends. To automate 20 Hz pulse detection, approaches such as template matching (also called matched filters) have been used^9,30^. Such hand-crafted algorithms require finding a compromise between the amount of detected pulses and their reliability, for instance only retaining pulses with an estimated signal to noise ratio (SNR) over 12 dB^9,18^. Moreover, the reliability of these approaches in more heterogeneous recording conditions is yet to be proven. When PAM stations are closer to the surface and/or to the coast, they are exposed to more noise^31^ (e.g. weather conditions, boat traffic) which hinders detection. Moreover, when recordings sessions are scarce, detection volume has to be optimized in order to get a sufficient amount of data for satisfactory statistical analysis. Machine learning can bring the necessary robustness to tackle these challenges^32,33^, with a data-driven approach able to cope with noise diversity and low SNR conditions.

Until now, no large scale analysis has been conducted on Mediterranean fin whale songs that could reveal the long-term evolution of their vocal behaviour. In this work we employ a CNN trained to screen recordings from multiple PAM stations in search of fin whale 20 Hz pulses. The results confirm the stereotypical IPIs observed in previous smaller scale analyses. Furthermore the data shows temporal variations such as inter-annual IPI shifts and a seasonal pulse frequency trend. These observations offer a more complete view of Western Mediterranean fin whales' vocal behaviour, and how it compares to that of other populations (or even other mysticete species).

## Material and methods

### Overview

**Figure 2:**
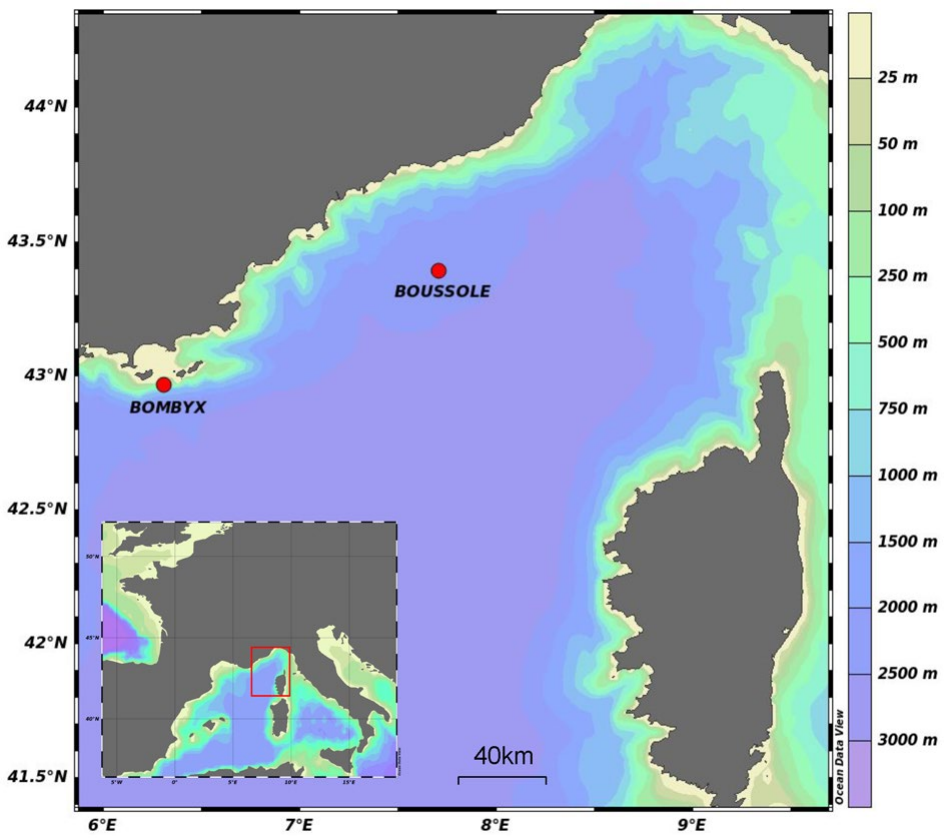
Map showing the two recording stations used in the analysis. This map was made using the Ocean Data View software^39^.

The acoustic material used in this study has been recorded at multiple PAM stations which have different recording devices. These were placed only several meters deep and relatively close to the coast (Table 1; Fig. 2) which may lead to high levels of noise exposure. Unlike the acoustic data from previous large-scale analyses^9,20^ the recordings from our setup present a wide range of noises that may hinder pulse detection. These conditions require methods which are resilient to sensor or environment diversity, and to acoustic masking and interference.

The methodology that we employed consists of three main steps. We first automatically detected fin whale 20 Hz pulses within the totality of our recordings using a machine learning model, producing a rough pulse timing estimate within $\sim$1 s of error. Then, a refined analysis of the spectro-temporal energy around the detection provided finer details about the timing, spectrum and SNR of each pulse. Eventually, we discarded estimations that were considered unreliable due to abnormal parameters or low SNR.

### Recorders

**Table 1:** Summary of the recording characteristics for each source. The data from Magnaghi was only used in the CNN training, not in the subsequent analysis.

Data source	Magnaghi^34^	Boussole^35^	Bombyx^36^	Total
Location	Tyrrhenian Sea	South of Sanremo	Port-Cros Island	Tyrrhenian Sea
Recording system	Magnaghi sono-buoy	EAR^37^	OSEAN-HNI Neptune	
Depth (m)	1	10-25	25	1-25
Recording year	1999	2008-2009	2015-2018	1999-2018
Sampling rate (kHz)	6	32	50	
ON/OFF protocole (min)	continuous	5/10	1/5 until Oct. 17, then 5/15	
Recorded time (h)	0.75	1752	3533	5286
Positive annotations	78	430	282	790
Negative annotations	396	4098	292	4786
Detection threshold		0.15	0.68	
Detected pulses		1418	2272	3 690
Detected A pulses		1182	1980	3162
Detected B pulses		292	236	523
Detected bouts		43	203	246

**Figure 3:**
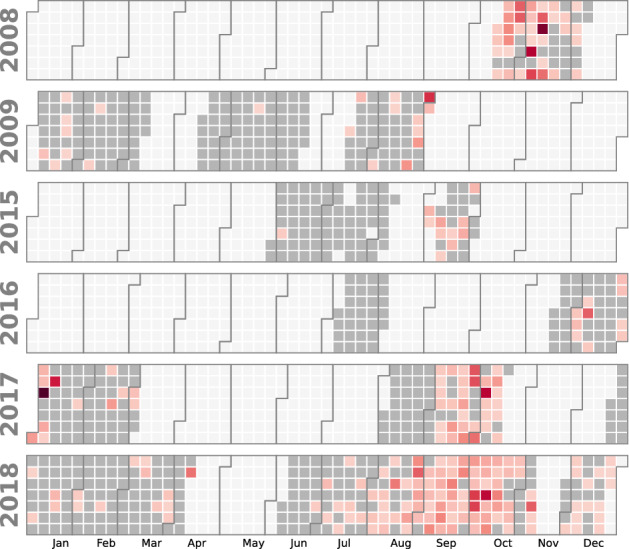
Calendar of the recorded days (grey cells). Shades of red denote the number of detected pulses normalized by the number of recorded hours (ranging from 0 to 30).

Recording characteristics for the three sources used in this study are summarized in Table 1, and detailed recording dates can be visualised in Fig. 3. The diversity of the data gathered poses great challenges for the automated analysis but also offers an opportunity for relatively robust performance measures, especially on the generalization capabilities of the machine learning models.

The different recording stations being in the same region (smaller than the range fin whales travel^38^), we consider the observed individuals to belong to the same population.

### Pulse detection

In order to process the 5286 h of recordings, we have developed a CNN that detects fin whale pulses in acoustic signals. The model was trained in a supervised fashion, with data labels gathered using an iterative process alternating between training, inference on unlabelled data and manual correction of predictions (active learning).

Starting from a single annotated song (see Magnaghi data in Table 1) a detection model was trained and then used for inference over all available recordings. Positive predictions were randomly sampled, manually corrected and added to the training set for the next iteration. By correcting model predictions, we mean adding false positives to the set of negative annotations and adding true positives to positive annotations. During the manual annotation/correction step, when a true pulse was encountered, the annotator searched for surrounding pulses and annotated them as well. This served to reduce the ‘iterative over-fitting’ effect, in which the model specializes to detect a specific type of pulse or pulses in a particular condition. This process of annotation, training and inference was repeated as a cycle until few or no manual corrections were required.

The resulting annotated database is described in Table 1, with the number of positive and negative samples for each data source. We detail below the procedures concerning the CNN.

#### Data pre-processing

During training, we prepare the input waveform by first selecting a 5 s window surrounding the annotation and downsampling it to 200 Hz (using the Fourier method). The pulses of interest are centered around 20 Hz with a bandwidth of up to 6 Hz (bandwidths are distributed differently between A and B pulses partly because of the sweep nature of the A pulse^19^, see Supplementary Figure 5). Therefore a Nyquist frequency of 100 Hz is sufficient to encode them. The waveform is then standardized to zero mean and unit variance, for lower variability in sound exposure level (SEL). To enforce generalization and better low SNR performances, we add synthetic brown noise to the input signals at around − 3 dB SNR. Brown noise was chosen for its similarity to the sea’s ambient noise.

We then compute the Mel spectrogram with a Hann window and Fast Fourier Transform (FFT) sizes of 256, a hop size of 32, and 128 logarithmically spaced frequency bins ranging from 0 to 100 Hz. While the Mel scale has been designed to mimic human listening characteristics, it is commonly used in the analysis of sound from other animals^40^ and remains useful for its logarithmic sampling on the frequency domain.

#### CNN model and training procedure

We designed a relatively low complexity CNN architecture (36 k parameters) that detects if a fin whale pulse is present in a given audio segment. It is composed of 3 depth-wise convolution layers^41^ with kernels of size 5 and strides of 1. The first two convolutions have 128 feature channels, while the last has only one (its output representing the probability of a pulse presence at a rate of 6.25 Hz).

The Mel spectrogram was compressed using $\log _{10}(1+ x\times 10^a)$ with $a$ being a trainable parameter of the model (inspired from Grill and Schlüter^40^). The 1D convolution layers were then applied along the temporal dimension, with frequency bins treated as distinct input features. We did not convolve on the frequency dimension since large frequency shifts are not expected in fin whale 20 Hz pulses. This does not impede the model from learning several pulse types with different spectral characteristics.

The two first convolutions are followed by batch normalization, leaky rectified linear unit activation and dropout ($p = 0.25$). Global maximum pooling and a sigmoid activation were applied after the last convolution. The network was trained as a binary classifier using a cross-entropy loss with targets indicating if the audio segment contains or not a fin whale pulse. We trained for 50 epochs with a batch size of 16, a learning rate of 0.001 (which decays by 3% at each epoch), an Adam optimizer^42^ and a weight decay L2 loss of 0.04. To cope with the imbalance of the two classes, positive examples were presented 4 times per epoch instead of 1 (over-sampling).

**Figure 4:**
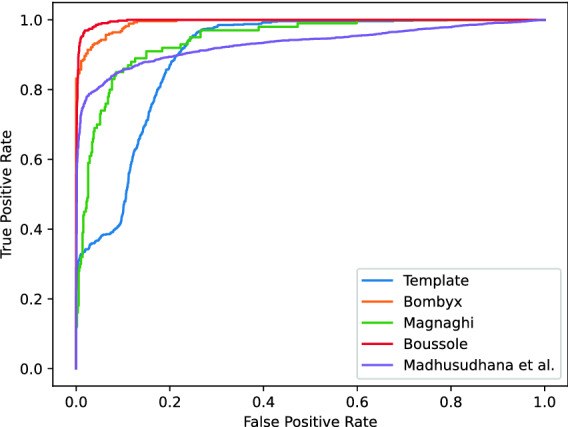
ROC curves for each test set (the two remaining sources serving as training set) and for the template matching method. The ROC curve of the model over the dataset published by Madhusudhana et al.^33^ is also displayed.

Hyper-parameters such as the number of features per layer or kernel sizes were selected using two of the sources for training and the third for testing, in a cross-fold manner (see Supplementary Figure 2). Figure 4 shows the receiving operating characteristics (ROC) curves of the selected architecture for each fold. The area under the ROC curves (AUC) are 0.992, 0.943, and 0.997 for Bombyx, Magnaghi and Boussole test sets respectively. We also evaluated the behaviour of the model when tested on signals at multiple SNR levels of added Brown noise (Supplementary Figure 4).

#### Model inference and validation

While the model was trained to detect pulse presence in 5 s segments, the convolutional stack is designed to maintain the temporal resolution of the predictions throughout the network. We thus discarded the last maximum pooling layer at the end of the CNN and retained as pulse times the highest predictions above a given detection threshold within sliding 4 s windows. These timings are approximate up to the size of the receptive field of the network (0.8 s).

We selected detection thresholds at the balance point of the ROC curves (equal sensitivity and specificity) for each data source separately (see Table 1). This choice gives a sensitivity/specificity of 0.96 for Bombyx and 0.97 for Boussole data. The Magnaghi data was not included in the subsequent analysis since multiple fin whale songs are overlapping in the available segments, and the proposed method does not cope with this.

We conducted two experiments to validate the pulse detection procedure: (1) comparison to a commonly used template matching method; (2) comparison to a state-of-the-art deep learning approach on an unseen dataset.

Automatic detection of mysticete sound events has commonly been performed using template matching (also known as matched filter) either in the time domain^9,30^, or in the time-frequency space^43,44^. We have compared this approach to our CNN-based detection system. Spectrograms of all the annotated pulses in the training set were averaged to produce a pulse template. We then applied varying thresholds on the cross-correlation product of samples with this template. The resulting detection performances are presented as a ROC curve in Fig. 4. The AUC of the template matching method is 0.898 (5 to 10 points less than the CNN model, depending on the train/test fold).

Furthermore, we report the performance of our CNN model on the dataset published by Madhusudhana et al.^33^, which also studies a CNN based fin whale 20 Hz pulse detection. The resulting area under the precision recall curve and peak F1-score are 0.96 and 0.88, when their reported best overall performances are 0.95 and 0.91 respectively (the comparison of scores is not entirely reliable since the published data is only a subset of the dataset in the paper’s experiments). We thus show that our model generalizes well to new data. Moreover we obtain comparable performances to an approach with 33% more parameters and which exploits the sequentiality of the pulses by using recurrent network layers (potentially introducing more complex inductive biases).

**Figure 5:**
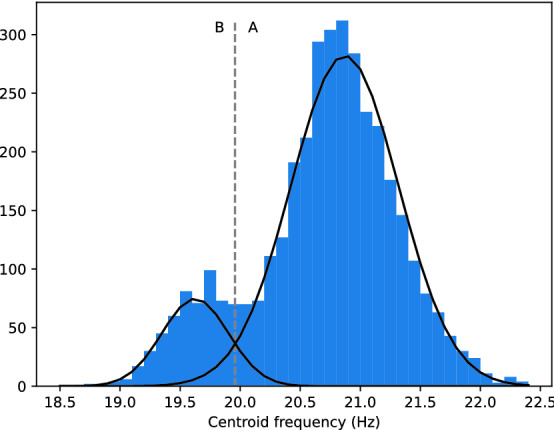
Histogram of the center frequencies of the detected pulses (post filtering). Black lines denote the fitted GMM, and the dotted line denotes the discrimination threshold between the two pulse types.

### Spectro-temporal pulse analysis

Following the detection process, the pulse analysis extracted a more detailed description of each pulse, comprising an exact time position, the center frequency, the bandwidth and the SNR.

For this analysis, we selected an 8 s window surrounding the prediction peak ($T=[0,8]$), applied a band-pass Butterworth filter of order 3 between 10 and 30 Hz, and resampled the waveform at 250 Hz. We computed a spectrogram (Hann window of 1024 including 75% of zero padding and 97% overlap) with spectral and temporal resolutions of 0.24 Hz and 0.03 s respectively. We started by estimating a precise time position of the pulse ${\hat{t}}$ by selecting the column of the maximum value in the 18–22 Hz frequency band (Eq. 1). This value was kept for the later IPI measurements.

1 $$\hat{t} = {\,\text{argmax}\,}_{t \in T}\left( \max _{f \in [18,22]}\left(\textbf {S}(f, t)\right) \right)$$

To measure the spectral envelope of the pulse, a 1.2 s window around ${\hat{t}}$ was max-pooled time wise. We withdrew background components to focus on the pulse spectra only, by subtracting an estimate of the background spectrum: the median of each frequency bin within the window $T$ (Eq. 2). Doing so, we mitigate effects such as impact of SNR on peak frequency and bandwidth, as observed by Helble et al.^10^.

2 $$\begin{aligned} E(f) = \max _{t \in [{\hat{t}}-0.6, {\hat{t}}+0.6]} \left( {\mathbf {S}}(f, t)\ \right) - \ \underset{t \in T}{\mathrm {median}}\ \left( {\mathbf {S}}(f, t)\right) \end{aligned}$$

The resulting pulse envelope was used to compute the left and right boundaries of the pulse spectrum, with $(\max _{f} E(f))/4$ as a threshold (equivalent to a − 6 dB bandwidth). Left and right intersection frequencies were linearly interpolated. The bandwidth then corresponds to the width, and the center frequency is the mid-point between these boundaries.

For later filtering by pulse quality, we estimated pulse SNR following Eq. 3. Pulse energy was computed as the maximum of its spectral envelope, and background energy as the median of the spectrogram surrounding the pulse.

3 $$\begin{aligned} E_{Background} = \underset{T \setminus [{\hat{t}}-1,{\hat{t}}+3]}{\underset{\ f \in [15, 25]}{\mathrm {median}}}\ {\mathbf {S}}(f, t), E_{Pulse} = \max _{f}E(f), SNR = 10\log _{10}\left( \frac{E_{Pulse}}{E_{Background}}\right) \end{aligned}$$

The pulse spectral characteristics of mysticetes are often described using the frequency of maximum energy (peak frequency) or the spectrum weighted mean (centroid frequency)^9,45^. We have chosen the center frequency as it appeared to be better suited for the discrimination between the two pulse types. In fact, when modeling the distribution of peak frequencies using a Gaussian mixture model, the two components (emerging from the two types of pulses) overlapped more than when using center frequencies. Indeed the Kullback–Leibler divergence between the Gaussian components in center frequency is significantly higher than that of peak frequencies (113 nats and 30 nats respectively).

Following the extraction of pulse characteristics, the IPI was computed as the difference between ${\hat{t}}$ of consecutive pulses. Pulses at a distance of less than 45 s were considered as being part of the same sequence, and sequences at a distance of less than 2 h were considered as being part of the same bout (following Watkins et al.^5^).

### Pre-analysis filtering

To filter out false positives, only pulses with a bandwidth below 6 Hz and a center frequency within $[18.5,22.5]$ were retained. Besides, only sequences with a mean SNR of at least 8 dB, and with at least 3 pulses were kept. Sequences containing IPIs below 11 s or above 45 s were discarded as well. The resulting number of registered pulses are shown in a calendar Fig. 3 and in Table 1.

To classify between A and B types, a two component Gaussian mixture model (GMM) was fitted on the center frequency data using the expectation-maximization (EM) algorithm (Fig. 5). This led to a threshold of 19.96 Hz to discriminate between the two types (Fig. 1). Even though the center frequency is found to evolve over time the change is sufficiently small to not interfere with the categorisation (Fig. 8).

### Temporal trends analysis

In order to conduct a temporal trend analysis, we need to extract points out of continuous distributions. This section describes the method employed in that regard, especially to extract stereotypical IPIs and center frequencies for varying temporal scales.

For each pulse type pair (‘AA’, ‘AB’, ‘BB’ and ‘BA’), the long-term evolution of stereotypical IPIs was analysed with an approach similar to Weirathmueller et al.^9^. From 2008 to 2018, for each 3 month period, the most frequent IPI was taken (IPIs were quantized to a resolution of 0.1 s). To withdraw periods with too few data for estimates to be reliable, only those with at least 100 pulse transitions were retained. Moreover, in order to gather only actual stereotypical IPIs, only those with a frequency of occurrence above 5% in their time period were kept.

In parallel, a study of intra-annual variations of pulse frequencies was conducted. For this statistical analysis, we quantized the center frequencies with a resolution of 0.1 Hz and grouped dates by months. Similarly than for IPIs, the most frequent center frequency of each month were retained, this time only for months with at least 200 pulses.

Once most frequent observations are gathered for each time period, linear least-squares regressions were conducted to estimate the linearity and the slope of measurements.

### Relationship between pulse frequency and IPI

Experiments were conducted to test the hypothesis from Weirathmueller et al.^9^ that the augmentation of the IPI through the years could be explained by the simultaneous decrease in pulse centroid frequencies. To dissociate this analysis from the link between pulse types and IPI, we fitted a 3 component Gaussian mixture model on the bi-dimensional representation of pulses (center frequency versus time until the next pulse). Thus, we were able to group the different pulse bi-grams (‘AA’, ‘AB’, and ‘BA’), and conduct a Pearson correlation analysis on each group independently. This bi-gram classification approach is relatively similar to the classification of units proposed by Archer et al.^17^ (combining spectrum and IPI).

## Results

The following results are taken from a database of 744 sequences with 3690 pulses in total (Table 1). This database is available online at http://sabiod.lis-lab.fr/pub/fin_whale_songs/.

### Stereotypical IPI

**Figure 6:**
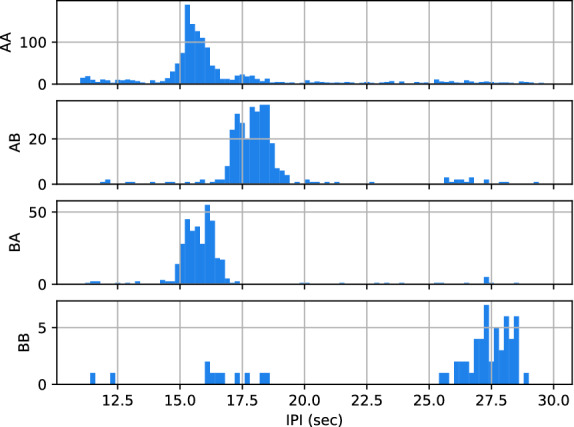
Histogram of the IPI for each type sequence (or bi-gram).

The time between a pair of consecutive pulses (IPI) appeared to be strongly determined by their type (Fig. 6). The typical interval for an ‘AB’ bi-gram was 2 s longer than that of ‘AA’ or ‘BA’. On the other hand, the ’BB’ pairs (less frequent but still commonly found) were 11 s longer on average.

**Figure 7:**
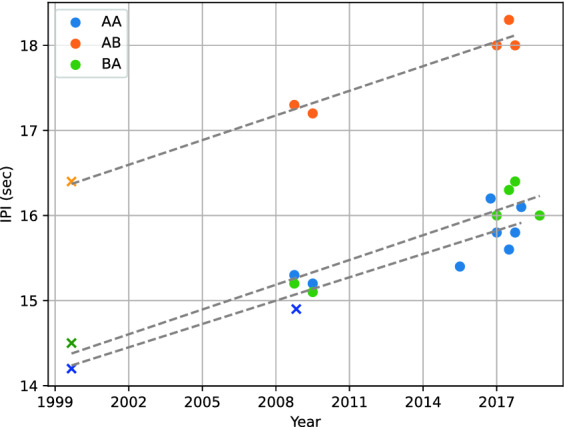
Scatter plot of the most frequent IPI per trimester for each type sequence. Fitted linear models are shown as grey dashed lines. Points extracted from Clark et al.^18^ and Castellote et al.^12^ appear as crosses.

These stereotypical IPIs showed a steady increase through time (Fig. 7). To increase the temporal window of this observation and place it among the literature on the Mediterranean fin whale song, we included measurements from previously published papers: the points measured in 1999 by Clark et al.^18^, and a point measured in 2008 by Castellote et al.^12^ (assuming it describes the most common pair ‘AA’, as it was not specified). The ‘BB’ sequence did not provide enough occurrences for the statistical tests to be relevant.

Linear models fitted on IPI growths of ‘AA’, ‘AB’ and ‘BA’ gave coefficients of determination of 0.86, 0.97, and 0.92 respectively (Fig. 7). The p-value for the null-hypothesis that the slope is not significantly different from 0 were all inferior to 0.001. The estimated slopes for the ‘AA’, ‘AB’, and ‘BA’ bi-grams are 0.091, 0.096, and 0.097 respectively (in s/year). This demonstrates a linear increase of all stereotypical IPIs by $\sim$ 0.1 s/year.

### Center frequency

**Figure 8:**
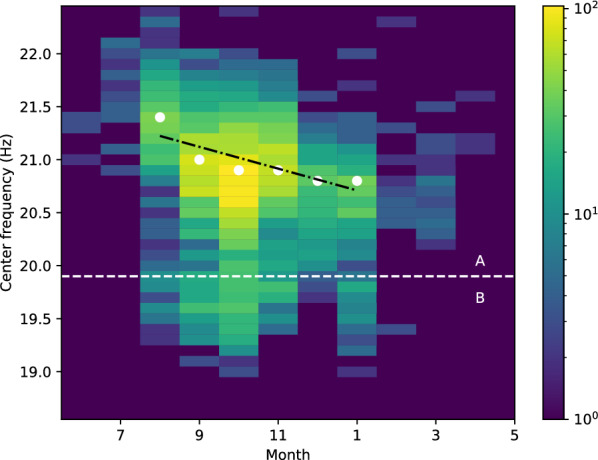
Bi-histogram of the center frequencies against months of the year. The horizontal line shows the separation between type A and type B pulses. The fitted linear model is shown as a black dashed line.

In a similar fashion, we also analysed the changes over time in spectral characteristics of pulses. We observed an intra-annual decrease in pulse center frequency between the months of August and February (Fig. 8). However no inter-annual trend was found (the Pearson analysis yielded a correlation coefficient of 0.05 between pulse absolute dates and their center frequency, also illustrated in Supplementary Figure 3).

Fitting a linear model on the intra-annual trend gave a coefficient of determination of 0.73, with an estimated slope of − 0.10 (in Hz/month, Fig. 8). The p-value for the null-hypothesis that the slope is not significantly different from 0 was 0.03.

We also show the distribution of type B pulses with respect to months of the year in Fig. 8, below the white dashed line. However, there is not enough data to draw an analysis like the one conducted for the type A pulses.

For comparison with other previous studies, we ran the same analysis using peak and centroid frequencies. The slope of the observed intra-annual trends were similar for all metrics (− 0.08 Hz/month, − 0.10 Hz/month, and − 0.13 Hz/month for peak, center, and centroid frequencies respectively).

### Correlation between center frequency and IPI

The observed Mediterranean fin whales stereotypical IPIs support the idea of a link between pulse frequency and IPI (as seen in Fig. 6, ‘AA’ shows the shortest IPI on average). This was originally stated by Weirathmueller et al.^9^ when simultaneously observing a yearly decrease in pulse frequency and a yearly increase in IPI. We further tested this hypothesis by analysing the correlation between IPI and center frequency (for pulses with IPIs between 14 and 20 s and for each bi-gram separately).

**Figure 9:**
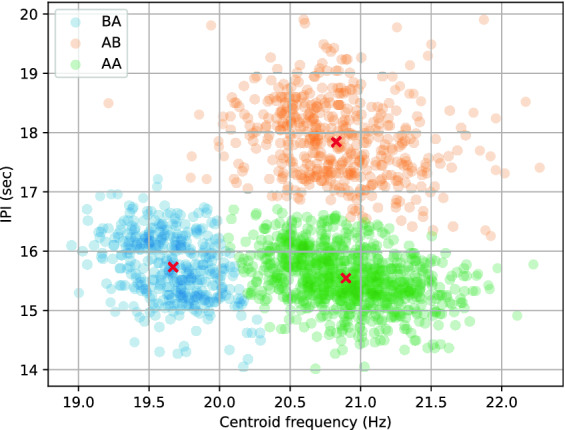
Scatter plot of pulses center frequency against the time until the next pulse (IPI). Colors denote the GMM assignation, whose means are marked with crosses.

Figure 9 shows the scatter plot of the pulses with their assignation to each Gaussian mixture component (one per pulse bi-gram). The Pearson analyses output correlation coefficients of − 0.37, − 0.22, and − 0.35 for ‘BA’, ‘AB’, and ‘AA’ bi-grams respectively (all p-values are below 0.001), which suggests no continuous relationship between the two variables.

## Discussion

The present study first proposes to use a small CNN architecture for fin whale 20 Hz pulse detection. It gives satisfactory performances when tested on four different test sets (three datasets annotated by us and one from another study^33^). The use of this CNN on the available data first led to the confirmation of the local stereotypical IPIs being determined by the pulse type sequence. These results were previously shown on relatively small corpora of around 100 pulses^18^, we confirm them with an order of magnitude larger corpus and in a span of 10 years.

**Table 2:** Summary of fin whale song trends studies. For intra-annual IPI shifts, since trends are not linear, we report the difference between low IPI season and high IPI season (summer vs winter). Note that the inter-annual IPI shift of Morano et al.^11^ is reported between two consecutive years only, and the frequency shifts of Leroy et al.^28^ are for the 99 Hz pulse rather than the 20 Hz pulse.

Study	Location	Inter-annual		Intra-annual	
		Frequency	IPI	Frequency	IPI
Weirathmueller et al.^9^	N.E. Pacific	− 0.17 Hz/year	0.5–0.9 s/year	–	–
Oleson et al.^21^	N Pacific	–	–	–	+ 7.5 s
Leroy et al.^28^	Indian	− 0.21 Hz/year	-	$\sim$ − 0.1 Hz/mth	-
Helble et al.^10^	N. Pacific	–	0.6–1.3 s/year	–	–
Morano et al.^11^	N.W. Atlantic	–	0.5 s/year	–	+ 5.5 s
Watkins et al.^5^	N.W. Atlantic	–	–	–	+ 6 s
Furumaki et al.^46^	Chukchi Sea	–	$\sim$ 0.5 s/year	–	$\sim$ + 1 s
Širović et al.^24^	Gulf of California	–	$\sim$ 1 s/year	–	$\sim$ + 8 s
Wood and Širović^20^	W. Antarctic	− 0.2 Hz/year	0.1 s/year	–	–
Ours	Mediterranean	–	0.1 s/year	–0.1 Hz/month	–

### Inter-annual shifts

We further show how these stereotypical IPIs evolve over the years, following a linear growth of approximately 0.1 s/year over the past 20 years. Such trends have been found in fin whales across the world’s oceans with different slopes^9–11,20,24,46^ (Table  2). Weirathmueller et al.^9^ state that the increasing IPI might be linked to the downward frequency shift, lower frequency pulses potentially being more demanding in energy. We measured a low correlation coefficient between the two variables, and our data did not show any evidence for an inter-annual center frequency decrease. These observations thus go against this hypothesis, but more data is required to draw firm conclusions.

As for the IPI shift slopes, differences across populations shown in Table 2 might arise culturally^9,10,20^. The cultural hypothesis holds if a phenomenon is shown to be learned and taught by peers, not genetically determined, and not triggered solely by environmental factors^47^. For humpback whales, the divergence of songs across populations may be due to local innovations^48^, but it seems less plausible for fin whales (change is steady over decades and slow enough to be unnoticeable to the whales^20^). Nonetheless, since local singing patterns drift independently, song conformity could operate at different rates (e.g. depending on population density) and explain the IPI shift slope differences.

Another plausible explanation for these differences across populations would be if the factor causing the IPI shifts operates at different rates. For instance, for the hypothesis of the post-whaling population recovery (increasing density and animal sizes), recovery rates could differ between Mediterranean and Pacific waters. McDonald et al.^26^ have hypothesized the increasing population size to be responsible for blue whale inter-annual song trends. In this case, the higher whale density would alter the sexually selected trade-off between call amplitude and call frequency. The Mediterranean fin whale population is growing^49,50^, but there is no obvious analogous amplitude/IPI trade-off that could explain a rise in IPI in response to an increasing population density.

In terms of inter-annual shifts in vocalization frequencies, they have been documented in several mysticete species such as blue whales^25,26,45^ and bowhead whales^51^. Fin whales also show such trends in the Pacific^9^, Antarctic^20^, and Indian^28^ oceans (Table  2). Numerous hypotheses have been formulated for the cause of this phenomenon, such as the increase in population density or body sizes (following the cessation of commercial whaling^26^), the increase in calling depth^27^, the augmentation of noise from melting icebergs^28^, or the acidification of the oceans affecting sound propagation^29^.

No inter-annual frequency shift was found in the analysed data: Mediterranean fin whales could be an exception to this widespread trend. Their isolation from populations for which this phenomenon was observed could explain this difference, in which case cultural implications would be suggested.

### Intra-annual shifts

Our data did not show any inter-annual decrease in vocalization frequency but rather an intra-annual decrease (− 0.10 Hz/month). Such phenomenon was previously observed in large mysticetes of the Indian Ocean including fin whales^28^, with a similar slope than we observed (Table  2). The latter study hypothesised pulse frequencies to follow seasonal ambient noise level variations (notably due to melting ice). Such phenomenon does not apply to the Mediterranean Sea.

On the other hand, studies of the Atlantic^5,11^ and Pacific^9,21^ oceans point to IPI increases during winter, before dropping back to autumn values (Table  2). This trend was hypothesised to be directly linked to the reproductive season^10,21^ (hormonal activity, progressive dilution of the competition). The same could apply to pulse frequency variations in the Mediterranean Sea, if we overhaul the observation by Notarbartolo-Di-Sciara et al.^1^ that there are no specific reproductive season for this population.

The calendar in Fig. 3 indeed seems to show an increased singing activity during autumn, but the unbalanced seasonal sampling through the years could cause an observational bias. Further work, perhaps requiring more data, is needed to precisely characterize the seasonal song trends in the Western Mediterranean Sea and confirm the existence of a frequency trend as well as the lack of IPI trend.

## Conclusion

Our study reveals the structure of the Mediterranean fin whale song from a statistical perspective, extending the previous analyses conducted for this population^12,18^. The CNN used for 20 Hz pulse detection showed robustness to data variability and state of the art performance. The approach relies on a relatively simple framework, sufficiently computationally efficient to be embedded in low-power micro-processors for applications such as real time alert systems for collision risk mitigation^52^.

This automatic approach enabled a long-term analysis revealing multi-year and seasonal trends in the Mediterranean fin whale song. Two parameters (IPI and pulse frequency) were analysed inter- and intra-annually. Table 2 summarises our findings along those of other studies. Such comparative results could contribute to understanding the factor(s) responsible for these trends observed worldwide, whether they are environmental, cultural, physiological, genomic or a combination thereof.

## Supplementary Information

Supplementary Information.

## Data Availability

All the data used in the analysis can be found at http://sabiod.lis-lab.fr/pub/fin_whale_songs/.
