# Author Correction: Temporal evolution of the Mediterranean fin whale song

**DOI:** 10.1038/s41598-022-25305-z

**Published:** 2022-12-02

**Authors:** Paul Best, Ricard Marxer, Sébastien Paris, Hervé Glotin

**Affiliations:** 1 Université de Toulon, Aix Marseille Univ, CNRS, LIS, DYNI, Marseille, France; 2 Pôle INPS, Marseille , France

Correction to: *Scientific Reports* 10.1038/s41598-022-15379-0, published online 09 August 2022

The original version of this Article contained errors.

Firstly, to increase its readability, Figure 1 was swapped with a black and white version of the image.

Consequently, the legend of Figure 1

“Spectrogram of a fin whale pulse sequence recorded by the Bombyx buoy in October 2018. Spectrogram parameters are described in section "Spectro-temporal pulse analysis". Dots show the center frequencies of the detected pulses, with white dashed lines showing the IPIs. The grey dashed line denotes the discrimination threshold between A and B pulse types, at 20 Hz.”

now reads:

“Spectrogram of a fin whale pulse sequence recorded by the Bombyx buoy in October 2018. Spectrogram parameters are described in section "Spectro-temporal pulse analysis". Dashes show the center frequencies of the detected pulses, with grey dashed lines indicating the IPIs. The blue dashed line denotes the discrimination threshold between A and B pulse types, at 20 Hz.”

Secondly, in Figure 8 the histogram did not accurately display the densest bins for the months of the year. Furthermore, the Figure wrongly scaled the data from 0 to 70 instead of from 10^0^ to 10^2^.

The original Figure 1 and 8 and accompanying legends appear below.

**Figure 1:**
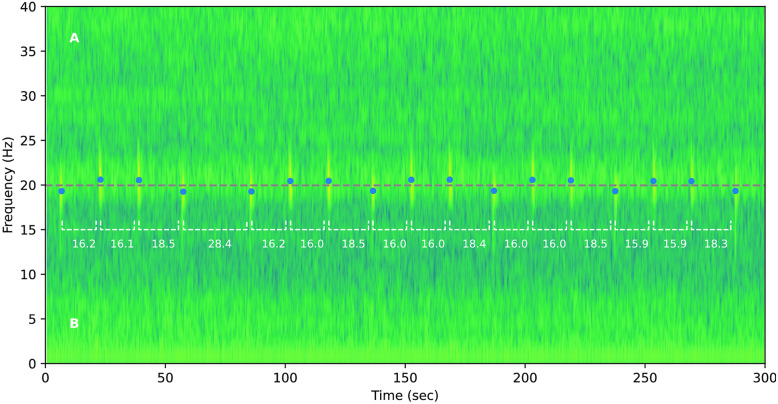
Spectrogram of a fin whale pulse sequence recorded by the Bombyx buoy in October 2018. Spectrogram parameters are described in section "Spectro-temporal pulse analysis". Dots show the center frequencies of the detected pulses, with white dashed lines showing the IPIs. The grey dashed line denotes the discrimination threshold between A and B pulse types, at 20 Hz.

**Figure 8:**
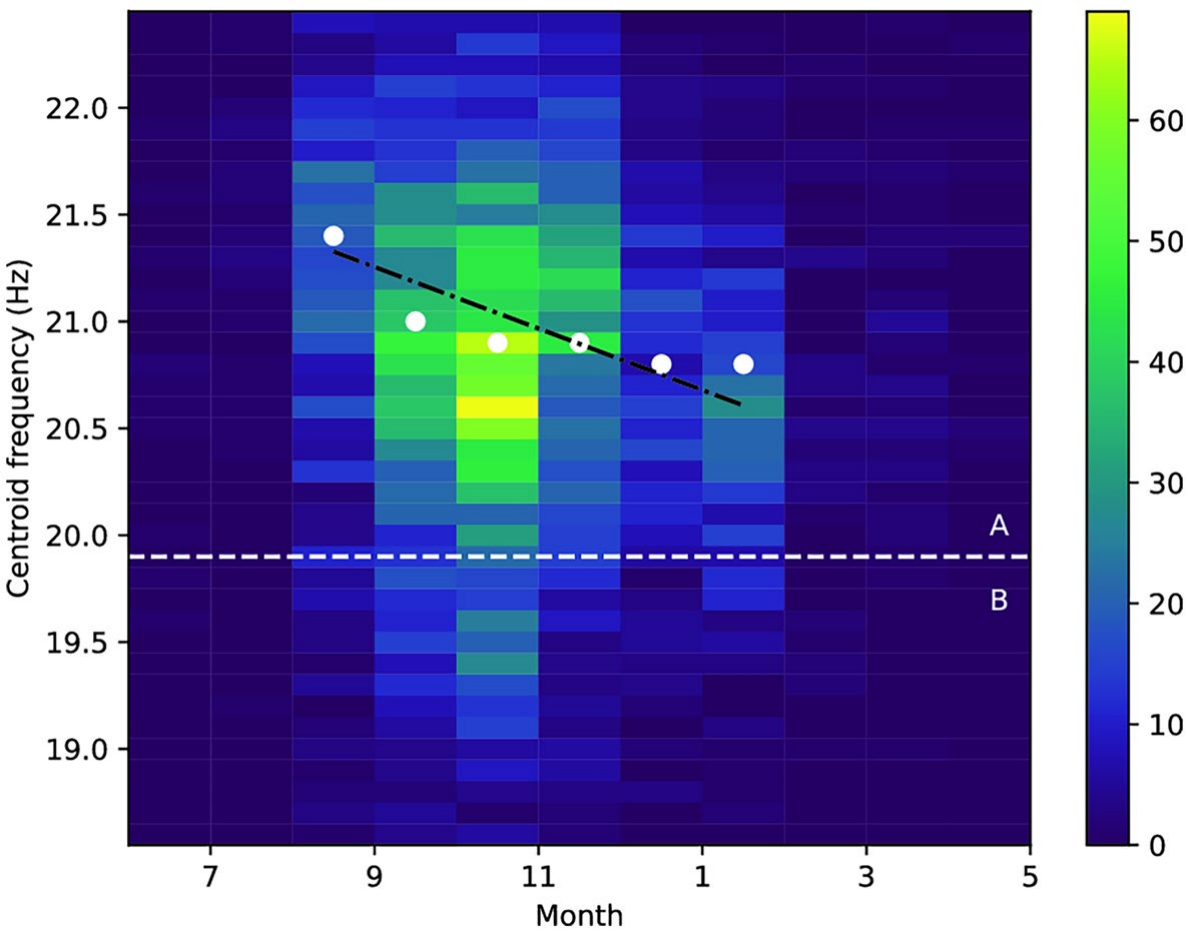
Bi-histogram of the center frequencies against months of the year. The horizontal line shows the separation between type A and type B pulses. The fitted linear model is shown as a black dashed line.

The original Article has been corrected.

