# Supplementary material for: Temporal evolution of the Mediterranean fin whale song
Source: Sci Rep. 2022 Aug 9;12:13565. doi: 10.1038/s41598-022-15379-0 (PMC9363496; doi:10.1038/s41598-022-15379-0)
Supplement: Supplementary file 1 — Supplementary Information. [file 41598_2022_15379_MOESM1_ESM.pdf]

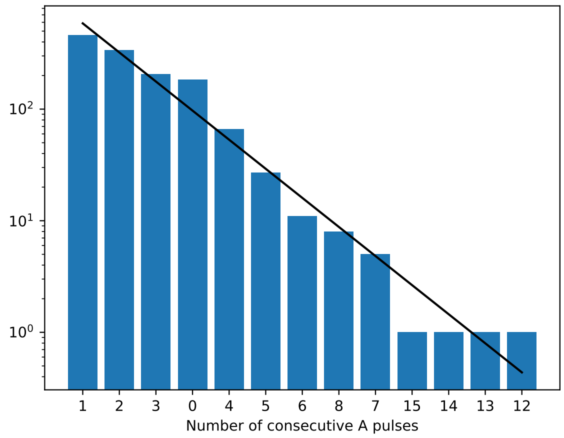

Supplementary Figure 1. Distribution of the number of type A pulses in between type B pulse. The black line denotes the fitted slope for PLC estimation.

1 Supplementary Material

1.1 Sequence analysis and Zipfs law

To our knowledge, most of the fin whale vocalization sequences are analysed as singlets, doublets or triplets[1–4], meaning a succession of the same pulse type, or an alternation between 2 pulse types. The Mediterranean fin whale songs apparently do not strictly follow those two patterns, but rather present a mixture of them. To analyse the sequences occurrence, with a system that is generic to their length, we thus use the number of consecutive A pulses (the type A pulse is chosen since it is much more frequent than the type B pulse). A singlet sequence 'AAAAA' thus becomes '5', a doublet sequence 'ABABABAB' becomes '1111', and a mixture sequence 'BAABBAAABABA' becomes '20311'. Such a system gives us insights on potential patterns and tendencies on the fin whale songs. We plot the histogram of the occurrences of these number of consecutive pulses in Figure 1, sorted from the most frequent to the least frequent.

Such a distribution seems appropriate for an estimation of the Zipf law power coefficient. Zipf law[5] is often used in language analysis, as it describes one common feature to all human languages. It is expressed by the following equation : $f \propto r^{-c}$, with f the frequency of a word, c the power law coefficient (PLC), and r the rank of the word (1 being the most frequent). The PLC describes how stereotyped the studied phenomenon is. When close to 0, the distribution is uniform, and each word has the same probability of occurrence. On the other hand, a high value of c means that a few words occur a lot when the others are rare. All human languages show a PLC of approximately 1, and any optimal communication canal would follow this characteristic (following the "principle of least effort").

Zipf's law has been used to characterize animal communication systems[6] on their potential language features. We thus fitted a linear model on the log frequencies against the log rank for our number of consecutive A pulses distribution (see Figure 1). The found PLC is 0.6, which can be interpreted as "the number of consecutive A pulses is more uniformly distributed than an optimal communication canal"[5].

References

1. Oleson, E. M., Širović, A., Bayless, A. R. & Hildebrand, J. A. Synchronous seasonal change in fin whale song in the north pacific. *PLoS ONE* **9**, e115678, DOI: 10.1371/journal.pone.0115678 (2014).
2. Morano, J. L. *et al.* Seasonal and geographical patterns of fin whale song in the western north atlantic ocean. *The J. Acoust. Soc. Am.* **132**, 1207–1212 (2012).
3. Helble, T. A. *et al.* Fin whale song patterns shift over time in the central north pacific. *Front. Mar. Sci.* **7**, 907 (2020).
4. Weirathmueller, M. J. *et al.* Spatial and temporal trends in fin whale vocalizations recorded in the ne pacific ocean between 2003-2013. *Plos one* **12**, e0186127 (2017).
5. Zipf, G. K. Human behaviour and the principle of least effort. (1950).
6. Kershenbaum, A. *et al.* Shannon entropy as a robust estimator of zipf's law in animal vocal communication repertoires. *Methods Ecol. Evol.* **12**, 553–564 (2021).

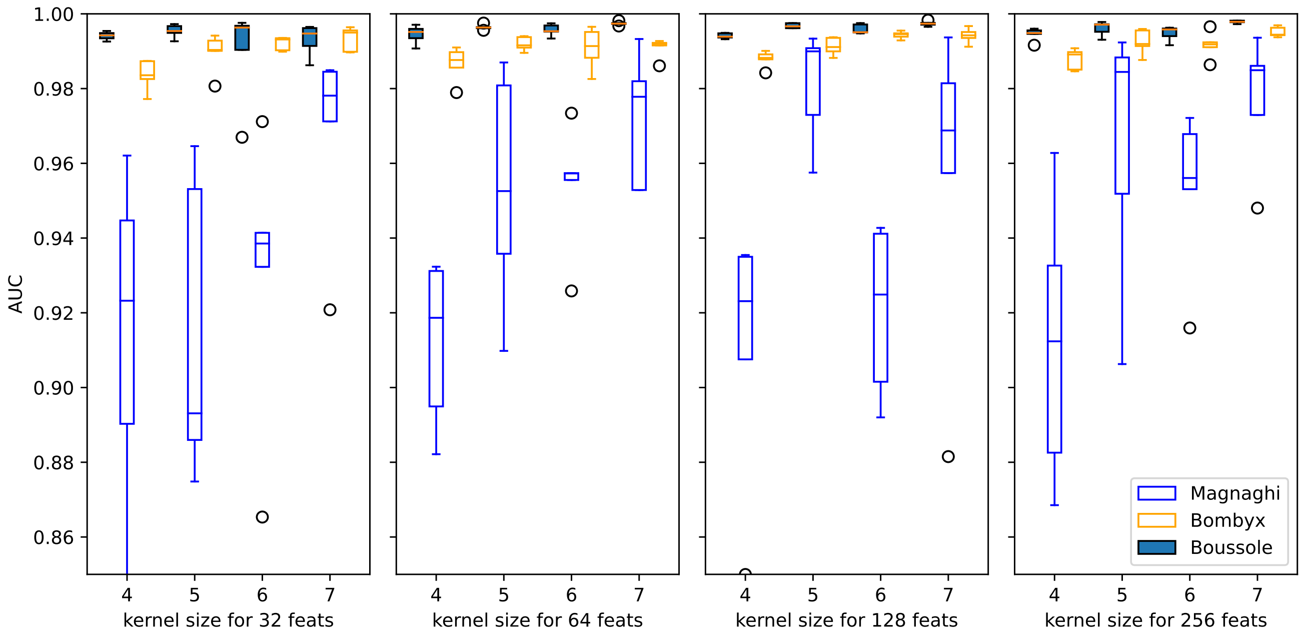

Supplementary Figure 2. Boxplots of the area under the receiving operating characteristics curve (AUC) of several combinations of hyper-parameters. For each number of features per layer, kernel size, and train/test fold, 5 runs were conducted. Folds are labelled with their test set (meaning that Bombyx scores report the performance of models trained on Magnaghi and Boussole).

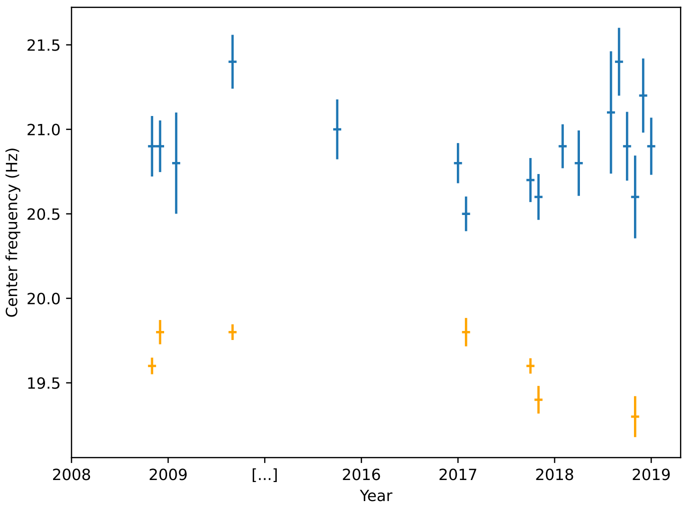

Supplementary Figure 3. Most frequent center frequencies for each month of the dataset (horizontal bars). Vertical bars denote the mean of the square difference with the given most frequent center frequency

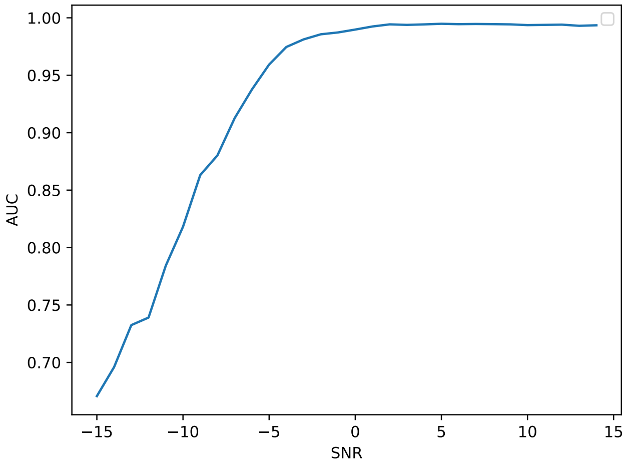

Supplementary Figure 4. AUC of the model as a function of the added brown noise level (measured in SNR as defined in Section 2.3.1)

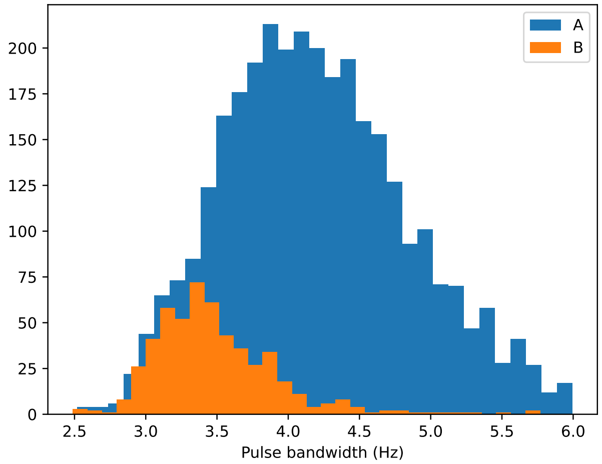

Supplementary Figure 5. Bandwidth of the detected pulses (at the peak energy -6dB), following the method described in Section 2.4
